# Amyloid-β and *APOE* genotype predict memory decline in cognitively unimpaired older individuals independently of Alzheimer’s disease polygenic risk score

**DOI:** 10.1186/s12883-022-02925-6

**Published:** 2022-12-15

**Authors:** Jori Tomassen, Anouk den Braber, Sven J. van der Lee, Lianne M. Reus, Elles Konijnenberg, Stephen F. Carter, Maqsood Yaqub, Bart N.M. van Berckel, Lyduine E. Collij, Dorret I. Boomsma, Eco J.C. de Geus, Philip Scheltens, Karl Herholz, Betty M. Tijms, Pieter Jelle Visser

**Affiliations:** 1 Alzheimer Center Amsterdam, Neurology, Vrije Universiteit Amsterdam, Amsterdam UMC location VUmc, Amsterdam, The Netherlands; 2 Amsterdam Neuroscience, Neurodegeneration, Amsterdam, The Netherlands; 3 Department of Biological Psychology, Amsterdam Public Health Research Institute, Vrije Universiteit Amsterdam, Amsterdam, The Netherlands; 4 Genomics of Neurodegenerative Diseases and Aging, Human Genetics, Vrije Universiteit Amsterdam, Amsterdam UMC location VUmc, Amsterdam, The Netherlands; 5 Wolfson Molecular Imaging Centre, Division of Neuroscience and Experimental Psychology, University of Manchester, Manchester, UK; 6 Department of Psychiatry, University of Cambridge, Cambridge, UK; 7 Department of Radiology & Nuclear Medicine, Vrije Universiteit Amsterdam, Amsterdam UMC location VUmc, Amsterdam, The Netherlands; 8 Amsterdam Neuroscience, Brain Imaging, Amsterdam, The Netherlands; 9 Alzheimer Center Limburg, School for Mental Health and Neuroscience, Maastricht University, Maastricht, the Netherlands; 10 Department of Neurobiology, Care Sciences and Society, Division of Neurogeriatrics, Karolinska Institutet, Stockholm, Sweden; 11 Alzheimer Center Amsterdam, Neurology, Amsterdam UMC location VUmc, 1007 MB Amsterdam, PO Box 7057, The Netherlands

**Keywords:** Preclinical Alzheimer’s disease, *APOE* genotype, Polygenic risk score, Amyloid-β, Cognitive decline, Neuropsychology, Longitudinal design

## Abstract

**Background:**

What combination of risk factors for Alzheimer’s disease (AD) are most predictive of cognitive decline in cognitively unimpaired individuals remains largely unclear. We studied associations between *APOE* genotype, AD-Polygenic Risk Scores (AD-PRS), amyloid-β pathology and decline in cognitive functioning over time in a large sample of cognitively unimpaired older individuals.

**Methods:**

We included 276 cognitively unimpaired older individuals (75 ± 10 years, 63% female) from the EMIF-AD PreclinAD cohort. An AD-PRS was calculated including 83 genome-wide significant variants. The *APOE* gene was not included in the PRS and was analyzed separately. Baseline amyloid-β status was assessed by visual read of [^18^F]flutemetamol-PET standardized uptake value images. At baseline and follow-up (2.0 ± 0.4 years), the cognitive domains of memory, attention, executive function, and language were measured. We used generalized estimating equations corrected for age, sex and center to examine associations between *APOE* genotype and AD-PRS with amyloid-β status. Linear mixed models corrected for age, sex, center and education were used to examine associations between *APOE* genotype, AD-PRS and amyloid-β status, and their interaction on changes in cognitive functioning over time.

**Results:**

Fifty-two participants (19%) had abnormal amyloid-β, and 84 participants (31%) carried at least one *APOE* ε4 allele. *APOE* genotype and AD-PRS were both associated with abnormal amyloid-β status. Increasingly more risk-full *APOE* genotype, a high AD-PRS and an abnormal amyloid-β status were associated with steeper decline in memory functioning in separate models (all p ≤ 0.02). A model including 4-way interaction term (*APOE*×AD-PRS×amyloid-β×time) was not significant. When modelled together, both *APOE* genotype and AD-PRS predicted steeper decline in memory functioning (*APOE* β(SE)=-0.05(0.02); AD-PRS β(SE)=-0.04(0.01)). Additionally, when modelled together, both amyloid-β status and AD-PRS predicted a steeper decline in memory functioning (amyloid-β β(SE)=-0.07(0.04); AD-PRS β(SE)=-0.04(0.01)). Modelling both *APOE* genotype and amyloid-β status, we observed an interaction, in which *APOE* genotype was related to steeper decline in memory and language functioning in amyloid-β abnormal individuals only (β(SE)=-0.13(0.06); β(SE)=-0.22(0.07), respectively).

**Conclusion:**

Our results suggest that *APOE* genotype is related to steeper decline in memory and language functioning in individuals with abnormal amyloid-β only. Furthermore, independent of amyloid-β status other genetic risk variants contribute to memory decline in initially cognitively unimpaired older individuals.

**Supplementary Information:**

The online version contains supplementary material available at 10.1186/s12883-022-02925-6.

## Background

Alzheimer’s disease (AD) is the most common cause of dementia. The development of AD starts with a preclinical stage where pathophysiological changes in brain amyloid-β are present, [1–4] while cognition is still intact. [5, 6] Once amyloid-β pathology is present it can take several years to decades to develop dementia. [7] AD drug development programs are shifting their focus to individuals in the earliest disease stage, because this is when individuals could benefit most from disease-modifying therapy. For this, it is important to be able to identify cognitively unimpaired individuals at risk of future cognitive decline in the ageing population.

Genetic risk factors might help to identify individuals with increased risk of developing AD. With heritability estimates of 58–79%, late-onset AD is a highly heritable disease. [8, 9] A well-known major genetic risk factor for AD is the *apolipoprotein E* (*APOE*) ɛ4 allele. *APOE* ɛ4 heterozygotes are at three-fold greater risk of AD and ɛ4 homozygotes at eight- to fifteen-fold compared to *APOE* ɛ4 non-carriers. [10] Additionally, it has been shown that *APOE* ɛ4 carriers have a higher prevalence of amyloid-β pathology at younger ages relative to non-carriers [5] and that *APOE* ɛ4 predicts cognitive decline in healthy controls. [11, 12] However, up to 40% of AD cases do not carry an *APOE* ɛ4 allele and the age at onset of AD dementia in *APOE* ɛ4 carriers varies widely, indicating that other genes or environmental risk factors are involved in causing the disease. Genome wide association studies (GWAS) in AD showed that, in addition to *APOE* ɛ4, over 80 genetic variants were significantly associated with AD. [13] By combining these multiple genetic variants, a so-called ‘polygenic risk score’ (PRS) can be calculated for each individual. Typically, higher PRS scores reflect increased risk for AD. [14] In cognitively unimpaired individuals, a high AD-PRS has been shown to be associated with amyloid-β pathology [15–18] and cognitive decline. [17–21] However, some studies did not find an association between AD-PRS and amyloid-β pathology [16] or cognitive decline. [22–24] Discrepant findings could be related to variability in the proportion of amyloid-β positive individuals in cognitively unimpaired groups, which ranges from 24 to 33%[5]. Other explanations for discordant results could be related to the number of genetic variants included in the AD-PRS, variability of cognitive tasks or composite scores used, or the follow-up duration. Furthermore, some studies included the *APOE* genotype in the AD-PRS, while other studies did not [16, 25–27]. Thus, it remains unclear to what extent the independent aspects of amyloid-β, *APOE* genotype and AD-PRS are related to cognitive decline in cognitively unimpaired individuals.

The aim of the present study was to investigate how *APOE* genotype, AD-PRS (*APOE* region excluded) and amyloid-β pathology relate to decline in cognition over time in cognitively unimpaired older individuals, and whether such effects are observed for specific cognitive domains (i.e., memory, attention, executive function and language).

## Methods

### Participants

Participants were part of the EMIF-AD PreclinAD study, which is a study investigating risk factors for amyloid-β pathology and cognitive decline in cognitively unimpaired older adults. [28] The PreclinAD study recruited participants from two sites: the Manchester and Newcastle Age and Cognitive Performance Research Cohort (ACPRC) in Manchester [29] and the Netherlands Twin Register (NTR) [30] in Amsterdam. Baseline inclusion criteria for the EMIF-AD PreclinAD study were age ≥ 60 years, Telephone Interview for Cognitive Status modified score of ≥ 23, [31] delayed recall score > − 1.5 SD of demographics-adjusted normative data on the Consortium to Establish a Registry for Alzheimer’s Disease 10-word list, [32] global Clinical Dementia Rating (CDR) score of 0, with a score of 0 on the memory sub domain [33] and a 15-item Geriatric Depression Scale score of < 11. [34] Baseline exclusion criteria were any neurological, systemic or psychiatric disorder that could cause cognitive impairment. Included participants had cognitive data available, an AD-PRS, an amyloid-β positron emission tomography (PET)-scan and/or *APOE* genotype data at their baseline visit (n = 276, of which 97 were complete twin pairs). All participants underwent an extensive baseline assessment including Mini-Mental State Examination (MMSE), neuropsychological assessment, acquiring years of education, and blood sampling. At two-year follow-up, MMSE, CDR and neuropsychological assessment were repeated, which were also used to monitor disease progression. According to protocol, if cognitive impairment was suspected by the study physician a neurologist was consulted and, if necessary or possible, a diagnostic work-up in the Alzheimer Center Amsterdam was performed. Each participant gave written informed consent, in accordance with the Medical Ethics Review Committee of the VU University Medical Center, the National Research Ethics Service Committee North West—Greater Manchester South and the Helsinki Declaration of 1975.

### Neuropsychological assessment

In Manchester, neuropsychological testing was performed during the research facility visit at both baseline and follow-up. In Amsterdam, neuropsychological testing at baseline was performed at home, and during follow-up at the VU University Medical Center or at home when participants were unable to come to the hospital (n = 19, 10%). We used the following tests to assess memory performance: the total immediate recall and delayed recall of the Rey Auditory Verbal Learning Test (RAVLT), [35, 36] the twenty minute recall of the Rey Complex Figure Test (RCFT), [37, 38] the total errors of the Cambridge Neuropsychological Test Automated Battery (CANTAB) Paired Associate Learning (PAL) test, [39, 40] and the total score of the Face Name Associated Memory Examination (FNAME) names and occupations delayed recall. [41, 42] To assess attention, we used the Trail Making Test (TMT) part A, [43] the forward condition of the Digit Span (total span score), [44] the simple accuracy score of the CANTAB Reaction Time (RTI) test and the mean response latency of the CANTAB Rapid Visual information Processing (RVP) test. [40] To test executive function, we used the TMT part B [43] (corrected for TMT part A), the backward condition of the Digit Span (total span score), [44] letter fluency (in English testing the letters F A S and in Dutch the letters D A T [45]) and the between errors score of the CANTAB Spatial-working Memory (SWM) test. [40] For language function, we used category fluency (animal fluency) one minute and the graded naming test (GNT). [46–49] FNAME data were missing in 22 participants (8%) at baseline and in 20 participants (9%) at follow-up. CANTAB RVP data were missing in 30 participants (11%) at baseline and in 31 participants (14%) at follow-up, due to lack of time or fatigue effects. For all other tests, 0-3% of test scores were missing.

### Genotyping and polygenic risk scoring

All genetic variants in our cohort were determined by applying standard genotyping and imputation methods and we applied established quality control methods. [50, 51] The genotype imputation method has been previously described. [52] In short, all individuals were genotyped using Illumina Global Screening array (GSA) with shared custom content (Illumina, Incl). We used high-quality genotyping in all individuals (individual call rate > 98%, variant call rate > 95%). All individuals’ reported sex matched with their genetic sex. Variants that departed from Hardy–Weinberg equilibrium were excluded at p < 1 × 10^− 6^. Genotypes were prepared for imputation using bcftools (v1.9) [53] for removing ambiguous (single nucleotide polymorphisms) SNPs, and flipping and swapping alleles to align to GRCh37/hg19. This was followed by haplotype phasing using SHAPEIT2 [54] and imputation of unobserved genotypes using Minimac3 [55] using a precompiled Haplotype Reference Consortium (HRC) reference panel. [56] We calculated a weighted individual AD-PRS based on the 83 genetic variants that showed genome-wide significant (GWS, p < 5e^− 8^) evidence of association with AD. [13]*APOE* haplotypes were not included in the AD-PRS. The selected variants were directly genotyped (median genotyping rate = 1) or imputed with high quality (median imputation score R² = 0.98, minimum R² = 0.5). The AD-PRS was generated by multiplying the genotype dosage of each risk allele for each variant by its respective weight and then summing across all variants. [57] Weights used can be found in (**Table S1**). [13] For analyses we used an ordinal *APOE* genotype variable (0 = *APOE* ɛ2ɛ2:ɛ2ɛ3; 1 = *APOE* ɛ3ɛ3; 2 = *APOE* ɛ2ɛ4:ɛ3ɛ4; 3 = *APOE* ɛ4ɛ4) and a normalized AD-PRS (mean = 0, standard deviation = 1). From all participants *APOE* data was missing for one participant (0.4%) and AD-PRS data was missing for five participants (1.8%). This study is independent of the discovery of the 83 genetic variants associated with AD, because participants were not included in Bellenguez et al. (2022).

### Amyloid-β PET-scan

At baseline, all participants were scanned from 90 to 110 min (4 × 5 min frames) after intravenous injection of 185 MBq (± 10%) [^18^F]flutemetamol. [28, 58] In Manchester, PET-scans were performed on a high-resolution research tomography brain scanner (HRRT; Siemens/CTI, Knoxville, TN, USA) at the Wolfson Molecular Imaging Centre of the University of Manchester. A 7-min transmission scan using a ^137^Cs point source was acquired for subsequent attenuation and scatter correction of the PET data. [59] In Amsterdam, PET-scans were performed on a Philips Ingenuity TF PET-MRI (Philips Healthcare, Cleveland, USA) at the Amsterdam UMC, location VU University Medical Center. Full acquisition details have been described previously. [28, 60] Immediately prior to each part of the PET-scan, a T1-weighted gradient echo pulse MRI scan was acquired for attenuation correction of the PET data. Three GE healthcare (GEHC) certified readers (SFC in Manchester; BNMvB and LEC in Amsterdam) visually rated standardized uptake value images as amyloid-β negative (predominantly white matter uptake) or positive (binding in one or more cortical brain region or striatum unilaterally), blinded to clinical and demographic data and according to GEHC guidelines. [61] Majority visual read was applied for both Manchester and Amsterdam obtained PET-scans.

### Statistical analysis

**Fig. 1:**
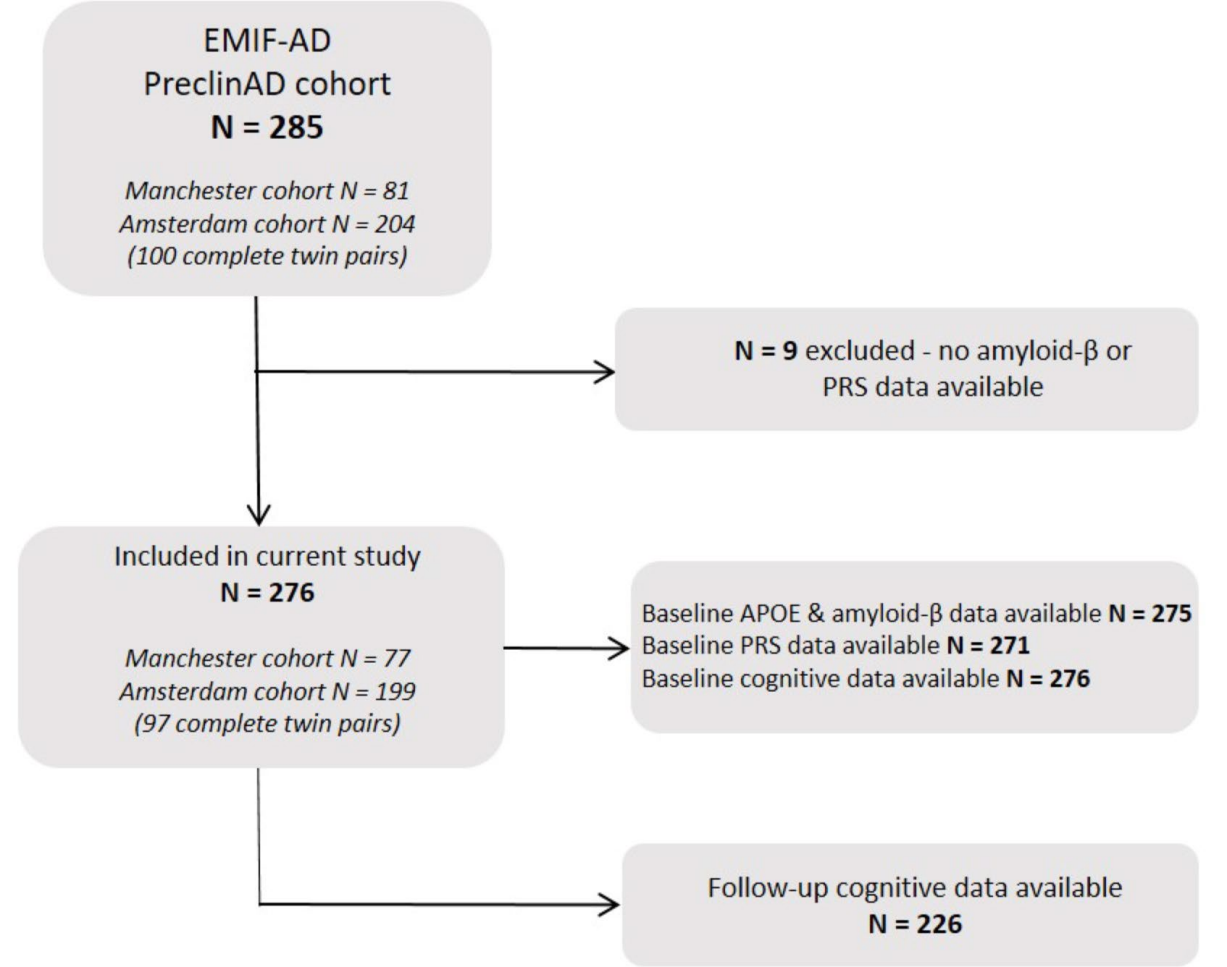
Flowchart of participant selection. None of the participants developed AD dementia or MCI between baseline and follow-up. Abbreviations: EMIF-AD = European Information Framework for Alzheimer’s Disease; amyloid-β = amyloid-beta; *APOE* = apolipoprotein E; PRS = polygenic risk score

We imputed missing cognitive test scores (0–14% of participants per test) by single imputation, using predictive mean matching as method [62] with follow-up time, genetic relatedness, baseline age, sex, education, amyloid-β status, and MMSE score as predictors. After imputation, baseline and two-year follow-up individual cognitive test scores were standardized using their baseline mean and standard deviation across the total sample (n = 285) (Fig. 1). CANTAB PAL, RVP and SWM scores and the TMT part A and B scores were inverted by multiplying the *z* score with − 1, so that for all tests lower scores reflect worse performance. Next, we averaged *z*-scores of the domain specific tests into four cognitive composite scores (i.e., memory, attention, executive function, and language).

First, we compared demographic variables between amyloid-β normal and amyloid-β abnormal groups using generalized estimating equations (GEE), including a random effect to account for family-relatedness in the Amsterdam cohort. We studied associations of *APOE* genotype (continuous dose: 0 = *APOE* ɛ2ɛ2:ɛ2ɛ3; 1 = *APOE* ɛ3ɛ3; 2 = *APOE* ɛ2ɛ4:ɛ3ɛ4; 3 = *APOE* ɛ4ɛ4) and AD-PRS (*z*-score) with amyloid-β status using separate GEE models (amyloid-β ~ *APOE*; amyloid-β ~ AD-PRS, respectively), including age, sex and center as covariates and *APOE* genotype and population substructure (principal components (PC) 1–3) depending on AD-PRS or *APOE* genotype as predictor. Next, we studied associations of *APOE* genotype (continuous dose), AD-PRS (*z*-score) and baseline amyloid-β status with changes in memory functioning over time using linear mixed effects models (LMMs). Models included subject-specific random intercepts and fixed slopes, a random effect for family, thereby correcting for clustering in the data, and were adjusted for age, sex, years of education and center. *APOE* genotype or population substructure (PC1-3) were added depending on using *APOE* genotype or AD-PRS as predictor. First, we tested with separate models the effects of *APOE* genotype, AD-PRS and amyloid-β status on memory decline with memory composite score as outcome (Memory ~ *APOE*×time; Memory ~ AD-PRS×time; Memory ~ amyloid-β×time, respectively). Next, we tested in separate models main and interaction effects of *APOE* genotype and AD-PRS on memory decline (Memory ~ *APOE*×AD-PRS×time), of *APOE* genotype and amyloid-β status on memory decline (Memory ~ *APOE*×amyloid-β×time) and AD-PRS and amyloid-β status (Memory ~ AD-PRS×amyloid-β×time). Finally, we tested the 4-way interaction of *APOE*×AD-PRS×amyloid-β×time on memory. Interaction terms were removed when not significant (*p* > 0.05). All models were repeated using cognitive composite scores of attention, executive function and language as outcome.

All analyses were corrected for multiple testing using the false discovery rate (FDR). FDR-corrected *p* values < 0.05 were considered significant. [63] Statistical analyses were performed in RStudio version 3.6.1 “Action of the Toes” (http://www.r-project.org/), using the following packages: mice_3.13.0, gee_4.13-20, lme4_1.1–26, lmerTest_3.1-2, emmeans_1.4.8, ggplot2_3.3.5.

## Results

### Demographics

We included 276 cognitively unimpaired older participants (Fig. 1) (mean age 74.7 ± 9.7, 63% female, 11.5 ± 2.7 years of education, mean follow-up 2.0 ± 0.4 years). Fifty-two participants (19%) had a positive visual read of their PET-scan. These participants were older, and had a lower composite memory, executive function and language score compared to participants with an amyloid-β negative PET-scan (Table 1).

**Table 1:** Baseline and follow-up sample characteristics

Baseline characteristics	Total sample	Amyloid normal	Amyloid abnormal
N Manchester cohort, n (%) Amsterdam cohort, n (%)	276 77 (27.9) 199 (72.1) (97 complete twin pairs)	218 49 (22.5) 169 (77.5) (76 complete twin pairs)	52 27 (51.9) 25 (48.1) (6 complete twin pairs)
Age, mean (SD) Age range	74.66 (9.65) 60.3–94.7	73.28 (9.47) 60.3–94.3	80.14 (7.66) * 62.9–94.7
Female, n (%)	174 (63.0)	134 (61.5)	37 (71.2)
Years of education, mean (SD)	11.47 (2.74)	11.56 (2.67)	11.24 (3.09)
Amyloid-β PET^$^, n (%)	270 (96.4)	218 (80.7)	52 (19.3)
*APOE* genotype, n (%)			
*e2e2*	2 (0.7)	2 (0.9)	0 (0)
*e2e3*	32 (11.6)	28 (12.8)	4 (7.7)
*e2e4*	9 (3.3)	7 (3.2)	2 (3.8)
*e3e3*	157 (56.9)	129 (59.2)	23 (44.2)
*e3e4*	68 (24.6)	49 (22.5)	18 (34.6)
*e4e4*	7 (2.5)	2 (0.9)	5 (9.6)
*APOE* ɛ4^#^ carrier, n (%)	84 (30.5)	58 (26.7)	25 (48.1)
Polygenic Risk Score for Alzheimer’s disease^^^ (*z* score), mean (SD)	0.00 (1.00)	-0.06 (1.00)	0.24 (0.99)
Neuropsychological testing, n (%)	274 (97.9)	216 (99)	52 (100)
MMSE, mean (SD)	28.9 (1.24)	29.0 (1.10)	28.5 (1.48) ^~^
Composite memory (*z* score), mean (SD)	-0.02 (0.71)	0.04 (0.69)	-0.23 (0.71) *
Composite attention (*z* score), mean (SD)	-0.25 (0.84)	-0.22 (0.85)	-0.34 (0.77)
Composite executive function (*z* score), mean (SD)	-0.44 (0.62)	-0.38 (0.59)	-0.67 (0.65) *
Composite language (*z* score), mean (SD)	-0.25 (0.81)	-0.17 (0.76)	-0.58 (0.95) *
Follow-up characteristics			
Follow-up time, mean (SD), y	2.04 (0.41)	2.01 (0.39)	2.16 (0.51)
Still in the study at follow-up, n (%)	226 (81.9)	184 (84.4)	41 (78.8)
Lost to follow-up	50 (18.1)	34 (15.6)	11 (21.2)

Baseline characteristics of the total sample. Data are presented as mean (SD), or n (%). ^$^PET data missing in 6 participants. ^#^*APOE* missing in 1 participant. ^^^Polygenic Risk Score missing in 5 participants. Amyloid groups were based on visual read [^18^F]flutemetamol PET standardized uptake value images. Abbreviations: amyloid-β = amyloid beta; *APOE* = *apolipoprotein* E; MMSE = Mini-mental state examination; PET = positron emission tomography. *p < 0.05^~^p > 0.05 < 0.10 different from amyloid normal PET group

### Associations of ***APOE*** genotype and AD-PRS with amyloid-β status

**Fig. 2:**
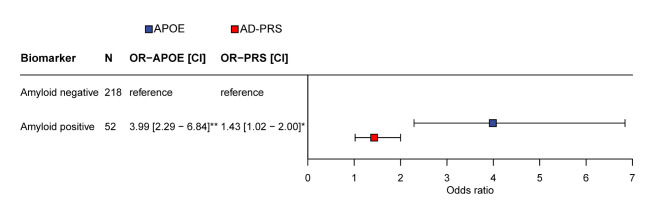
Associations between *APOE* genotype, AD-PRS and amyloid-β status. Values given are odds ratio [95% confidence interval] as estimated by Generalized estimating equations (predictor: *APOE* genotype or normalized AD-PRS; outcome: amyloid-β status (reference = amyloid-β negative status), including random effect for family-relatedness (comparable with logistic regressions, but corrected for clustering in the data), adjusted for age, sex, center, and population substructure (PC1-3). The odds ratio for the AD-PRS reflects the odds of having an amyloid-β positive status per one standard deviation increase in the AD-PRS. Amyloid-β status was based on visual read [^18^F]flutemetamol PET standardized uptake value images. Abbreviations: AD = Alzheimer’s Disease; *APOE* = *apolipoprotein E*; CI = confidence interval; OR = odds ratio; PC = principal components; PRS = polygenic risk score

Using separate models, we found that both higher *APOE* dose scores and higher AD-PRS were associated with a higher probability of amyloid-β positivity (odds ratio [OR] 3.99 [95% confidence interval (CI) 2.29 to 6.84], per allele; OR 1.43 [95% CI 1.02 to 2.00], respectively) (Fig. 2).

### Associations of ***APOE*** genotype, AD-PRS and amyloid-β with cognitive functioning

**Fig. 3:**
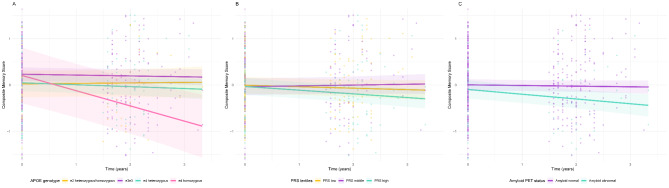
Effects of *APOE* genotype, AD-PRS and amyloid-β status on changes in memory composite scores. Model-based estimation of longitudinal changes in memory composite score, based on *APOE* genotype (A), polygenic risk score (PRS) for Alzheimer’s Disease (B) and amyloid-β status (C). Neuropsychological tests were *z*-transformed and averaged for the composite memory score. *APOE* genotype dosage variable used as factor for plots: ɛ2 heterozygous/homozygous (*APOE* ɛ2ɛ2:ɛ2ɛ3), ɛ3ɛ3 (*APOE* ɛ3ɛ3), ɛ4 heterozygous (*APOE* ɛ2ɛ4:ɛ3ɛ4) and ɛ4 homozygous (*APOE* ɛ4ɛ4). AD-PRS depicted in tertiles. Amyloid-β status was based on visual read [^18^F]flutemetamol PET standardized uptake value images. Fixed-effect covariates were baseline age, sex, education, center and population substructure. For statistics see Table 2. AD = Alzheimer’s disease; *APOE* = *apolipoprotein* E; PET = positron emission tomography; PRS = polygenic risk score

**Fig. 4:**
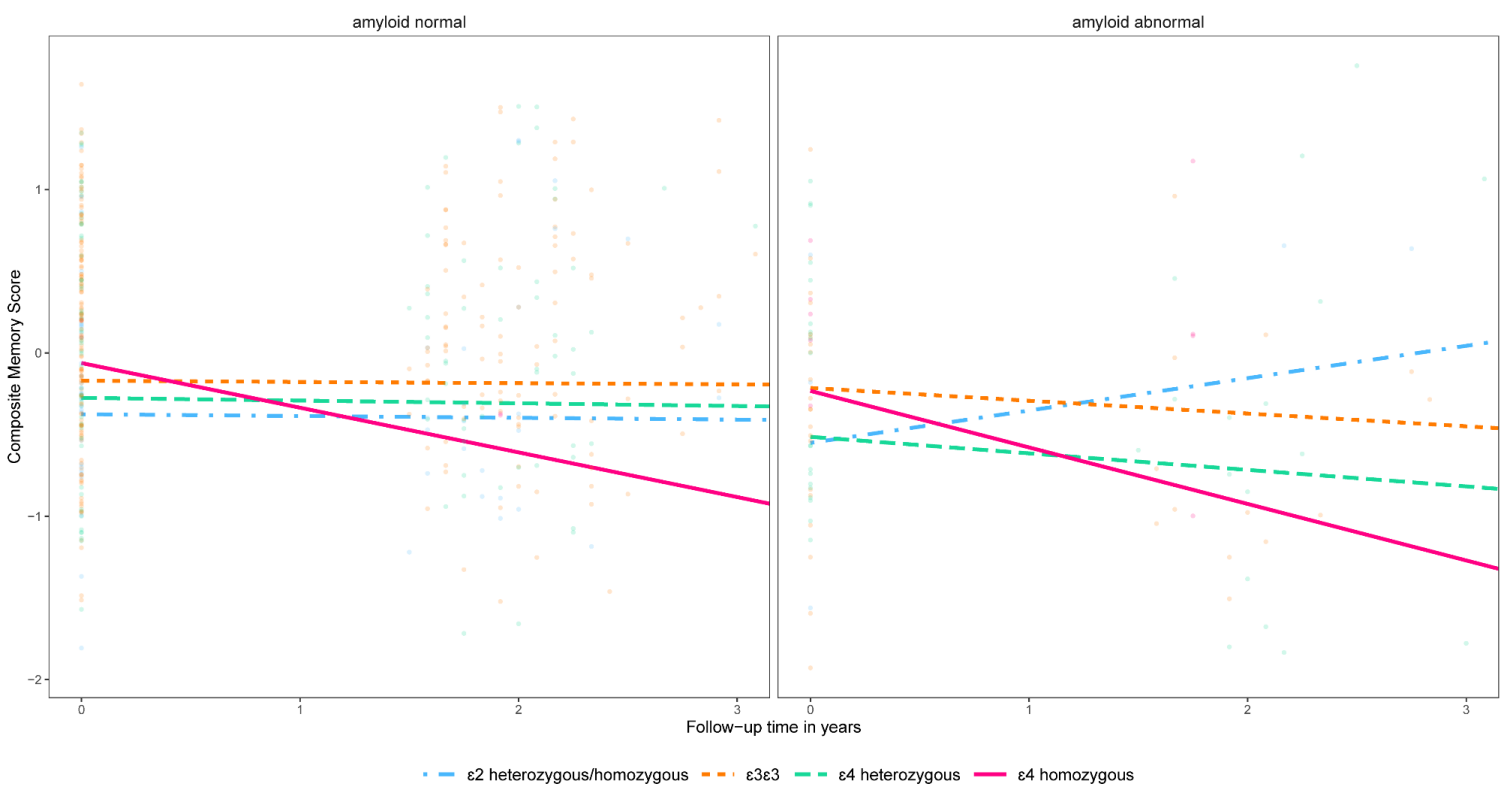
Interaction effects between *APOE* genotype and amyloid-β status with memory functioning over time. Amyloid-β status was based on visual read [^18^F]flutemetamol PET standardized uptake value images. *APOE* dosage variable used as factor for plots: ɛ2 heterozygous/homozygous (*APOE* ɛ2ɛ2:ɛ2ɛ3, n = 34), ɛ3ɛ3 (*APOE* ɛ3ɛ3, n = 157), ɛ4 heterozygous (*APOE* ɛ2ɛ4:ɛ3ɛ4, n = 77) and ɛ4 homozygous (*APOE* ɛ4ɛ4, n = 7). Neuropsychological tests were z-transformed and averaged for the composite memory score. Interaction effect was statistically significant, see Table 2. Abbreviations: *APOE* = *apolipoprotein* E

At baseline, no significant associations were observed between *APOE* genotype, AD-PRS and amyloid-β status with memory performance. Across the total group, memory scores declined over two years (β(SE) = -0.03(0.01), p = 0.04). Higher *APOE* dose was associated with steeper decline in memory functioning (β(SE) = -0.05(0.02), p = 0.008; Table 2; Fig. 3 A). Individuals with a high AD-PRS showed steeper decline in memory functioning over time than individuals with low AD-PRS (β(SE) = -0.04(0.01), p = 0.006; Table 2; Fig. 3B). Individuals with abnormal amyloid-β showed steeper decline in memory functioning over time than individuals with normal amyloid-β (β(SE) = -0.08(0.04), p = 0.02; Table 2; Fig. 3 C). We then examined the interactions of *APOE* genotype and AD-PRS on the rate of memory decline, which was not significant. Repeating analyses including *APOE* genotype and AD-PRS as main effects in the same model, we found that both higher *APOE* genotype dosage and a high AD-PRS were associated with steeper decline in memory over time (Table 2). When examining interactions of *APOE* genotype dosage and amyloid-β status on the rate of memory decline, we found a significant interaction effect (p-interaction = 0.02) (Fig. 4). Repeating analyses after stratifying for amyloid-β status, we found that a higher *APOE* dose was associated with steeper memory decline in individuals with abnormal amyloid-β (β(SE) = -0.13(0.06), p = 0.03), and no association was found in individuals with normal amyloid-β (β(SE) = -0.02(0.02), p = 0.46; Table 2). When examining interactions of AD-PRS and amyloid-β status on the rate of memory decline, no significant effects were found. Repeating analyses including AD-PRS and amyloid-β status as main effects in the same model, we found that both abnormal amyloid-β and high AD-PRS were related to steeper decline in memory over time (Table 2). The model including the 4-way interaction term (*APOE*×AD-PRS×amyloid-β×time) was not significant. After removing ɛ2ɛ4 carriers from the sample, the results and interpretation did not change (**Table S2**).

**Table 2:** Baseline and annual change effects of *APOE* genotype, PRS and amyloid-β status in memory composite scores

Composite memory score				
*Model*	*Fixed effects* *Baseline and interaction effects with time*	*β (SE)*	*p*	*pFDR*
Model 1	*APOE* *APOE* × time	-0.02 (0.07) -0.05 (0.02)	0.82 0.008	0.97 0.01
Model 2	AD-PRS AD-PRS × time	-0.002 (0.05) -0.04 (0.01)	0.97 0.006	0.97 0.01
Model 3	Amyloid-β Amyloid-β × time	-0.12 (0.10) -0.08 (0.04)	0.23 0.02	0.72 0.02
Model 4	*APOE* × AD-PRS *APOE* × AD-PRS × time	-0.13(0.07) 0.002 (0.02)	0.07 0.94	0.72 0.94
	*APOE* AD-PRS *APOE* × time AD-PRS × time	-0.03 (0.07) 0.002 (0.05) -0.05 (0.02) -0.04 (0.01)	0.66 0.97 0.01 0.008	0.97 0.97 0.03 0.03
Model 5	*APOE* × Amyloid-β *APOE* × Amyloid-β × time	-0.11 (0.15) -0.12 (0.05)	0.47 0.02	0.93 0.047
	Amyloid-β negative group: *APOE* *APOE* × time Amyloid-β positive group: *APOE* *APOE* × time	0.06 (0.08) -0.02 (0.02) -0.04 (0.18) -0.13 (0.06)	0.44 0.46 0.82 0.03	0.93 0.46 0.97 0.0495
Model 6	AD-PRS × Amyloid-β AD-PRS × Amyloid-β × time	-0.12 (0.10) 0.02 (0.04)	0.24 0.64	0.72 0.94
	AD-PRS Amyloid-β AD-PRS × time Amyloid-β × time	0.004 (0.05) -0.15 (0.10) -0.04 (0.01) -0.07 (0.04)	0.94 0.14 0.01 0.04	0.97 0.72 0.03 0.05

Relationship between *APOE* genotype dosage, AD-PRS, and baseline amyloid-β status and longitudinal memory performance. Linear Mixed Models with subject specific random intercepts and fixed slopes, corrected for age, sex, education and center. *APOE* genotype or PC1-3 were added depending on using *APOE* genotype or AD-PRS as predictor. Amyloid-β status was based on visual read [^18^F]flutemetamol PET standardized uptake value images. Abbreviations: amyloid-β = amyloid-beta; *APOE* = *apolipoprotein* E; FDR = False Discovery Rate; p = p value; PRS = polygenic risk score; PC = principal components; SE = standard error

Examining composite attention, executive function and language scores, we found across the whole group all scores improved over two years (attention: β(SE) = 0.05(0.02), p = 0.02; executive function: β(SE) = 0.06(0.02), p = 0.004; language: β(SE) = 0.11(0.02), p < 0.001). However, individuals with higher *APOE* dose showed steeper decline in language functioning (β(SE) = -0.07(0.03), p = 0.04; **Table S3, Figure S1G**). When examining interactions of *APOE* genotype and AD-PRS as main effects in the same model, we found that higher *APOE* genotype dosage was associated with steeper decline in language over time, while AD-PRS was not (**Table S3**). We then examined interactions of *APOE* genotype dosage and amyloid-β status on the rate of language decline, and we found a significant interaction effect (p-interaction = 0.003) (**Figure S2**). Repeating analyses after stratifying for amyloid-β status, we found that higher *APOE* dose was associated with steeper language decline in individuals with abnormal amyloid-β (β(SE) = -0.22(0.07), p = 0.002), and no association was found in individuals with normal amyloid-β (β(SE) = -0.02(0.04), p = 0.62; **Table S3**). No significant associations were found for *APOE* genotype, AD-PRS and amyloid-β status with attention and executive function performance over time.

## Discussion

In a cognitively unimpaired older population, we found that both higher *APOE* genotype dosage and higher AD-PRS were related to amyloid-β abnormality. Furthermore, we observed in separate models that AD-PRS, *APOE* genotype dosage and abnormal amyloid-β were associated with steeper decline in memory functioning over time in separate models. The association of higher *APOE* genotype dosage with steeper memory decline was dependent on amyloid-β abnormality, whereas AD-PRS explained variance in memory decline over time independently of *APOE* genotype dosage and amyloid-β status. In addition, *APOE* genotype dosage was associated with steeper decline in language functioning over time, and this association was dependent on amyloid-β abnormality. These results suggest that amyloid-β status, *APOE* genotype and AD-PRS all explain parts of cognitive decline in older individuals with initially normal cognition.

Our findings that both higher *APOE* genotype dosage and higher AD-PRS were associated with amyloid-β pathology replicates previous findings in cognitively unimpaired individuals. [64, 65] This suggests that besides *APOE* ɛ4 other genes may contribute to amyloid-β abnormality. Pathway-enrichment analyses [13] of the SNPs that were included in the AD-PRS showed most significant genes to be related to amyloid-β and tau, but also to lipid metabolism and immunity, highlighting the role of microglia and its potential interaction with amyloid precursor protein (APP) metabolism in AD development. [66]

At baseline, none of the variables examined were associated with memory performance as measured by a composite consisting of five memory tests. We previously did find an association between abnormal amyloid-β and lower baseline RCFT 3-minute recall scores in the Amsterdam cohort of the EMIF-AD preclinAD study. [67] Combining the two EMIF-AD cohorts in the sample of this study, using a composite memory score that combines multiple tests instead of separate tests and assessing associations with LMMs instead of GEEs may explain the different findings. When examining memory performance over time, we observed that an abnormal amyloid-β status was related to steeper decline in memory functioning over time compared to a normal amyloid-β status, which is in line with previous preclinical AD studies. [68–70] We also found that both a high AD-PRS and high *APOE* genotype dosage was related to decline in memory functioning over time. This observation is largely consistent with earlier studies in cognitively unimpaired individuals. [18, 19, 22, 24, 71] We further extend the literature by showing that *APOE* genotype dosage and high AD-PRS explained variance in memory decline independently. Further research is needed to understand whether specific risk genes contribute to the rate of cognitive decline in preclinical AD. While the effect of high *APOE* genotype dosage was specific for individuals with abnormal amyloid-β (which is in line with a previous study [72]), the high AD-PRS was related to steeper memory decline independently of amyloid-β status. Possibly, some SNPs included in the AD-PRS reflect risk for cognitive decline, through pathways which might be unrelated to amyloid-β pathology. Previous studies using pathway analysis methods on AD GWAS results showed that genetic variants linked to AD risk were associated with the immune system, [73] endocytosis, cholesterol metabolism, amyloid-β clearance and tau metabolism. [74, 75] Future research should investigate if pathway-specific PRSs contribute to distinct aspects of cognitive decline in AD. Additionally, *APOE* genotype dosage was associated with decline in language performance over time, dependent on amyloid-β abnormality. We previously showed in cognitively unimpaired individuals that the very first cognitive changes in the early stages of AD are to be found in memory and language domains. [76] As all cognitive scores improved over two years, reflecting learning effects, and we found no relation between *APOE* genotype, AD-PRS and amyloid-β status and decline on attention and executive function, it is possible with a longer follow-up period these individuals may start to show decline in these domains as well. Furthermore, our study evaluated genetic risk factors for AD development while previous population studies indicated that environmental factors also influence dementia risk. [77] We previously showed environmental factors to contribute to onset of amyloid-β aggregation, but not particularly to cognitive decline. [76] For future research, it will be of interest to investigate the contribution of risk genes and their possible interaction with environmental risk factors to AD development.

A strength of the present study is the relatively large sample of cognitively unimpaired older individuals with a substantial age range (60–94), AD biomarkers, genetic risk data, and repeated neuropsychological assessment available. Possible limitations are that by using a dichotomous variable for amyloid-β burden, more sensitive information could have been lost, because binary visual reads disregard the potential significance of quantifying early, pathological, amyloid-β accumulation. [78] In relation to *APOE* genotypes, we placed both *APOE* ɛ2ɛ4 and ɛ3ɛ4 carriers into the same category of the *APOE* genotype variable even though previous studies have shown that ɛ3ɛ4 carriers may have worse outcomes than ɛ2ɛ4 carriers. [79] However, removing ɛ2ɛ4 carriers from our sample did not change the results nor interpretation (**Table S2**). Since it has been shown that predictive performance of AD-PRS based on European samples is lower in non-European ancestry samples, [80] our results might not be directly generalizable due to the fact our study was solely based on a European sample. Although AD-PRS was associated with steeper memory decline in our study, effect sizes were small, which may limit its potential clinical use. Additionally, none of the participants progressed to mild cognitive impairment or dementia yet. In this study, 50 participants did not complete follow-up assessment and their current cognitive status is unknown. Comparing their baseline characteristics to remaining individuals in the study showed that they were older and were more often female and *APOE* ɛ4 carriers compared to the 226 individuals who were assessed at follow-up (see **Table S4**). The follow-up duration of an average of two years may have been too short to observe decline or clinical progression in initially cognitively unimpaired individuals, who take on average 5–6 years to show decline or clinical progression. [81] For this reason we aim to continue following our cohort.

## Conclusion

Our findings provide further support among cognitively unimpaired older individuals that, in addition to abnormal amyloid-β and *APOE* genotype, the AD-PRS is also associated with memory decline. Future research should further investigate which specific genetic variants contribute to memory decline and through which mechanism, because this will be important for developing treatments that may prevent decline in cognition.

## Electronic supplementary material

Below is the link to the electronic supplementary material.

Supplementary Material 1: Figure S1.

Supplementary Material 2: Figure S2.

Supplementary Material 3: Supplementary Materials.

## Data Availability

The datasets used and/or analysed during the current study are available from the corresponding author on reasonable request and after signing a material transfer agreement.

## Abbreviations

AD: Alzheimer’s disease
*APOE*: *apolipoprotein E*
APP: amyloid precursor protein
CANTAB: Cambridge Neuropsychological Test Automated Battery
CI: confidence interval
FNAME: Face Name Associated Memory Examination
GEE: generalized estimating equations
GEHC: GE Healthcare
GSA: Global Screening array
GWAS: Genome Wide Association Studies
LMM: linear mixed effects models
MMSE: Mini-Mental State Examination
OR: odds ratio
PAL: Paired Associate Learning
PC: Population Substructure
PET: positron emission tomography
PRS: Polygenic Risk Scores
RAVLT: Rey Auditory Verbal Learning Test
RCFT: Rey Complex Figure Test
SNPs: single nucleotide polymorphisms

## References

[1] Jack CRJ (2013). Lancet Neurology.

[2] Gordon BA (2018). Spatial patterns of neuroimaging biomarker change in individuals from families with autosomal dominant Alzheimer’s disease: a longitudinal study. Lancet Neurol.

[3] Bateman RJ (2012). Clinical and biomarker changes in dominantly inherited Alzheimer’s disease. N Engl J Med.

[4] Tomlinson BE, Blessed G, Roth M (1968). Observations on the brains of non-demented old people. J Neurol Sci.

[5] Jansen WJ (2022). Prevalence Estimates of Amyloid Abnormality Across the Alzheimer Disease Clinical Spectrum. JAMA Neurol.

[6] Donohue MC (2017). Association Between Elevated Brain Amyloid and Subsequent Cognitive Decline Among Cognitively Normal Persons. JAMA.

[7] Sperling RA, et al. Toward defining the preclinical stages of Alzheimer’s disease: Recommendations from the National Institute on Aging-Alzheimer’s Association workgroups on diagnostic guidelines for Alzheimer’s disease. Alzheimer’s & dementia. 2011;7.3: 280-292. 10.1016/j.jalz.2011.03.003 PMC3220946 21514248

[8] Gatz M (2006). Role of genes and environments for explaining Alzheimer disease. Arch Gen Psychiatry.

[9] Dubois B (2016). Preclinical Alzheimer’s disease: definition, natural history, and diagnostic criteria. Alzheimer’s Dement.

[10] Corder EH (1993). Gene dose of apolipoprotein E type 4 allele and the risk of Alzheimer’s disease in late onset families. Science.

[11] Caselli RJ (2007). Cognitive domain decline in healthy apolipoprotein E ε4 homozygotes before the diagnosis of mild cognitive impairment. Arch Neurol.

[12] Lim YY (2015). APOE ε4 moderates amyloid-related memory decline in preclinical Alzheimer’s disease. Neurobiol Aging.

[13] Bellenguez C, et al. New insights into the genetic etiology of Alzheimer’s disease and related dementias. Nature genetics. 2022:1–25. 10.1038/s41588-022-01024-z PMC9005347 35379992

[14] Scheltens P (2021). Alzheimer’s disease. The Lancet.

[15] Ebenau JL (2021). Risk of dementia in APOE ε4 carriers is mitigated by a polygenic risk score. Alzheimer’s & Dementia: Diagnosis Assessment & Disease Monitoring.

[16] Darst BF (2017). Pathway-specific polygenic risk scores as predictors of amyloid-β deposition and cognitive function in a sample at increased risk for Alzheimer’s disease. J Alzheimers Dis.

[17] Mormino EC (2016). Polygenic risk of Alzheimer disease is associated with early- and late-life processes. Neurology.

[18] Porter T (2018). Utility of an Alzheimer’s disease risk-weighted polygenic risk score for predicting rates of cognitive decline in preclinical Alzheimer’s disease: a prospective longitudinal study. J Alzheimers Dis.

[19] Carrasquillo MM (2015). Late-onset Alzheimer’s risk variants in memory decline, incident mild cognitive impairment, and Alzheimer’s disease. Neurobiol Aging.

[20] Skoog I, et al. A non-APOE polygenic risk score for Alzheimer’s disease is associated with CSF neurofilament light in a representative sample of cognitively unimpaired 70-year-olds. The Journals of Gerontology: Series A (2021). 10.1093/gerona/glab030 PMC8140047 33512503

[21] Marden JR (2016). Using an Alzheimer’s Disease polygenic risk score to predict memory decline in black and white Americans over 14 years of follow-up Running head: AD polygenic risk score predicting memory decline. Alzheimer Dis Assoc Disord.

[22] Verhaaren BF (2013). Alzheimer’s disease genes and cognition in the nondemented general population. Biol Psychiatry.

[23] Andrews SJ, Das D, Cherbuin N, Anstey KJ, Easteal S (2016). Association of genetic risk factors with cognitive decline: the PATH through life project. Neurobiol Aging.

[24] Riaz M (2021). Effect of APOE and a polygenic risk score on incident dementia and cognitive decline in a healthy older population. Aging Cell.

[25] Ge T (2018). Dissociable influences of < em > APOE</em > ε4 and polygenic risk of AD dementia on amyloid and cognition. Neurology.

[26] Tan CH (2018). Polygenic hazard score: an enrichment marker for Alzheimer’s associated amyloid and tau deposition. Acta Neuropathol.

[27] Cruchaga C (2018). Polygenic risk score of sporadic late-onset Alzheimer’s disease reveals a shared architecture with the familial and early-onset forms. Alzheimer’s Dement.

[28] Konijnenberg E (2018). The EMIF-AD PreclinAD study: study design and baseline cohort overview. Alzheimers Res Ther.

[29] Rabbitt PMA (2004). The University of Manchester Longitudinal Study of Cognition in Normal Healthy Old Age, 1983 through 2003. Aging Neuropsychol Cognition.

[30] Willemsen G (2013). The Adult Netherlands Twin Register: twenty-five years of survey and biological data collection. Twin Res Hum Genet.

[31] de Jager CA, Budge MM, Clarke R (2003). Int J Geriatr Psychiatry.

[32] Morris, JC, et al. The consortium to establish a registry for Alzheimer’s disease (CERAD): I. Clinical and neuropsychological assessment of Alzheimer’s disease. Neurology. 1989 10.1212/wnl.39.9.1159 2771064

[33] Morris, JC. Current vision and scoring rules the clinical dementia rating (CDR). Neurology. 1993;43:2412-2414. 10.1212/wnl.43.11.2412-a 8232972

[34] Yesavage JA (1982). J Psychiatr Res.

[35] Saan R, Deelman B De 15-woordentest A en B (een voorlopige handleiding). *Groningen: Afdeling Neuropsychologie, AZG* (1986).

[36] Rey A L’examen clinique en psychologie [Clinical psychological examination] Presses Universitaires de France. *Paris, France* (1964).

[37] Meyers JE, Bayless JD, Meyers KR (1996). Appl Neuropsychol.

[38] Snitz BE (2013). Cognitive trajectories associated with beta-amyloid deposition in the oldest-old without dementia. Neurology.

[39] Reijs BLR (2017). Memory Correlates of Alzheimer’s Disease Cerebrospinal Fluid Markers: A Longitudinal Cohort Study. J Alzheimers Dis.

[40] Robbins TW (1994). Cambridge Neuropsychological Test Automated Battery (CANTAB): a factor analytic study of a large sample of normal elderly volunteers. Dement Geriatr Cogn Disord.

[41] Papp KV (2014). Development of a Psychometrically Equivalent Short Form of the Face-Name Associative Memory Exam for use Along the Early Alzheimer’s Disease Trajectory. Clin Neuropsychologist.

[42] Rentz, DM, et al. Face-name associative memory performance is related to amyloid burden in normal elderly. Neuropsychologia. 2011;49.9:2776-2783. 10.1016/j.neuropsychologia.2011.06.006 PMC3137730 21689670

[43] Reitan, RM. Validity of the Trail Making Test as an indicator of organic brain damage. Perceptual and motor skills. 1958;8.3:271-276.

[44] Wechsler, D. Wechsler adult intelligence scale-revised (WAIS-R). Psychological Corporation. 1981.

[45] Schmand B, Groenink SC, van den Dungen M (2008). [Letter fluency: psychometric properties and Dutch normative data]. Tijdschr Gerontol Geriatr.

[46] Lindeboom J, Schmand B, Tulner L, Walstra G, Jonker C (2002). J Neurol Neurosurg Psychiatry.

[47] McKenna P, Warrington EK (1980). J Neurol Neurosurg Psychiatry.

[48] Van der Elst W, Van Boxtel MPJ, Van Breukelen GJP, Jolles J (2006). The Stroop Color-Word Test: Influence of Age, Sex, and Education; and Normative Data for a Large Sample Across the Adult Age Range. Assessment.

[49] Bird CM, Papadopoulou K, Ricciardelli P, Rossor MN, Cipolotti L (2004). Monitoring cognitive changes: Psychometric properties of six cognitive tests. Br J Clin Psychol.

[50] Bos I (2018). The EMIF-AD Multimodal Biomarker Discovery study: design, methods and cohort characteristics. Alzheimers Res Ther.

[51] Hong S (2020). Genome-wide association study of Alzheimer’s disease CSF biomarkers in the EMIF-AD Multimodal Biomarker Discovery dataset. Translational Psychiatry.

[52] Tesi N (2020). Immune response and endocytosis pathways are associated with the resilience against Alzheimer’s disease. Translational psychiatry.

[53] Narasimhan V (2016). BCFtools/RoH: a hidden Markov model approach for detecting autozygosity from next-generation sequencing data. Bioinformatics.

[54] Delaneau O, Marchini J, Zagury J-F (2012). A linear complexity phasing method for thousands of genomes. Nat Methods.

[55] Das S (2016). Next-generation genotype imputation service and methods. Nat Genet.

[56] McCarthy S, et al Haplotype Reference, C.(2016). A reference panel of 64,976 haplotypes for genotype imputation. *Nat Genet* **48**, 1279–1283. 10.1038/ng.3643 PMC5388176 27548312

[57] Dudbridge F (2013). Power and predictive accuracy of polygenic risk scores. PLoS Genet.

[58] Nelissen N (2009). Phase 1 Study of the Pittsburgh Compound B Derivative 18F-Flutemetamol in Healthy Volunteers and Patients with Probable Alzheimer Disease. J Nucl Med.

[59] Sibomana M, et al. in *IEEE Symposium Conference Record Nuclear Science* 2004. 2647–2651 (IEEE).

[60] Collij L (2018). Assessing Amyloid Pathology in Cognitively Normal Subjects using [(18)F]Flutemetamol PET: Comparing Visual Reads and Quantitative Methods. J Nucl Med.

[61] GEHealthcare. EPAR product information - summary of product characteristics. (2014).

[62] Van Buuren S, Groothuis-Oudshoorn K (2011). mice: Multivariate imputation by chained equations in R. J Stat Softw.

[63] Benjamini Y, Hochberg Y (1995). Controlling the False Discovery Rate: A Practical and Powerful Approach to Multiple Testing. J Roy Stat Soc: Ser B (Methodol).

[64] Sleegers K (2015). A 22-single nucleotide polymorphism Alzheimer’s disease risk score correlates with family history, onset age, and cerebrospinal fluid Aβ42. Alzheimer’s Dement.

[65] Lopresti BJ (2020). Influence of apolipoprotein-E genotype on brain amyloid load and longitudinal trajectories. Neurobiol Aging.

[66] Bellenguez C, et al New insights on the genetic etiology of Alzheimer’s and related dementia. *MedRxiv* (2020).

[67] Konijnenberg E (2019). Association of amyloid pathology with memory performance and cognitive complaints in cognitively normal older adults: a monozygotic twin study. Neurobiol Aging.

[68] Mortamais M (2017). Detecting cognitive changes in preclinical Alzheimer’s disease: A review of its feasibility. Alzheimer’s Dement.

[69] Doraiswamy PM (2012). Amyloid-β assessed by florbetapir F 18 PET and 18-month cognitive decline: a multicenter study. Neurology.

[70] Landau SM (2012). Amyloid deposition, hypometabolism, and longitudinal cognitive decline. Ann Neurol.

[71] De Jager PL (2012). A genome-wide scan for common variants affecting the rate of age-related cognitive decline. Neurobiol Aging.

[72] Mormino EC (2014). Amyloid and APOE ε4 interact to influence short-term decline in preclinical Alzheimer disease. Neurology.

[73] International Genomics of Alzheimer’s Disease (2015). Convergent genetic and expression data implicate immunity in Alzheimer’s disease. Alzheimers Dement.

[74] Kunkle BW (2019). Genetic meta-analysis of diagnosed Alzheimer’s disease identifies new risk loci and implicates Aβ, tau, immunity and lipid processing. Nat Genet.

[75] Sims R, Hill M, Williams J (2020). The multiplex model of the genetics of Alzheimer’s disease. Nat Neurosci.

[76] Tomassen J (2022). Abnormal cerebrospinal fluid levels of amyloid and tau are associated with cognitive decline over time in cognitively normal older adults: A monozygotic twin study. Alzheimer’s & Dementia: Translational Research & Clinical Interventions.

[77] Livingston G (2020). Dementia prevention, intervention, and care: 2020 report of the Lancet Commission. The Lancet.

[78] Villeneuve S (2015). Existing Pittsburgh Compound-B positron emission tomography thresholds are too high: statistical and pathological evaluation. Brain.

[79] Liu C-C, Kanekiyo T, Xu H, Bu G (2013). Apolipoprotein E and Alzheimer disease: risk, mechanisms and therapy. Nat Reviews Neurol.

[80] Duncan L (2019). Analysis of polygenic risk score usage and performance in diverse human populations. Nat Commun.

[81] Vermunt L (2019). Duration of preclinical, prodromal, and dementia stages of Alzheimer’s disease in relation to age, sex, and APOE genotype. Alzheimer’s Dement.

