# Supplementary figures and images for: Amyloid-β and APOE genotype predict memory decline in cognitively unimpaired older individuals independently of Alzheimer’s disease polygenic risk score
Source: BMC Neurol. 2022 Dec 15;22:484. doi: 10.1186/s12883-022-02925-6 (PMC9753236; doi:10.1186/s12883-022-02925-6)

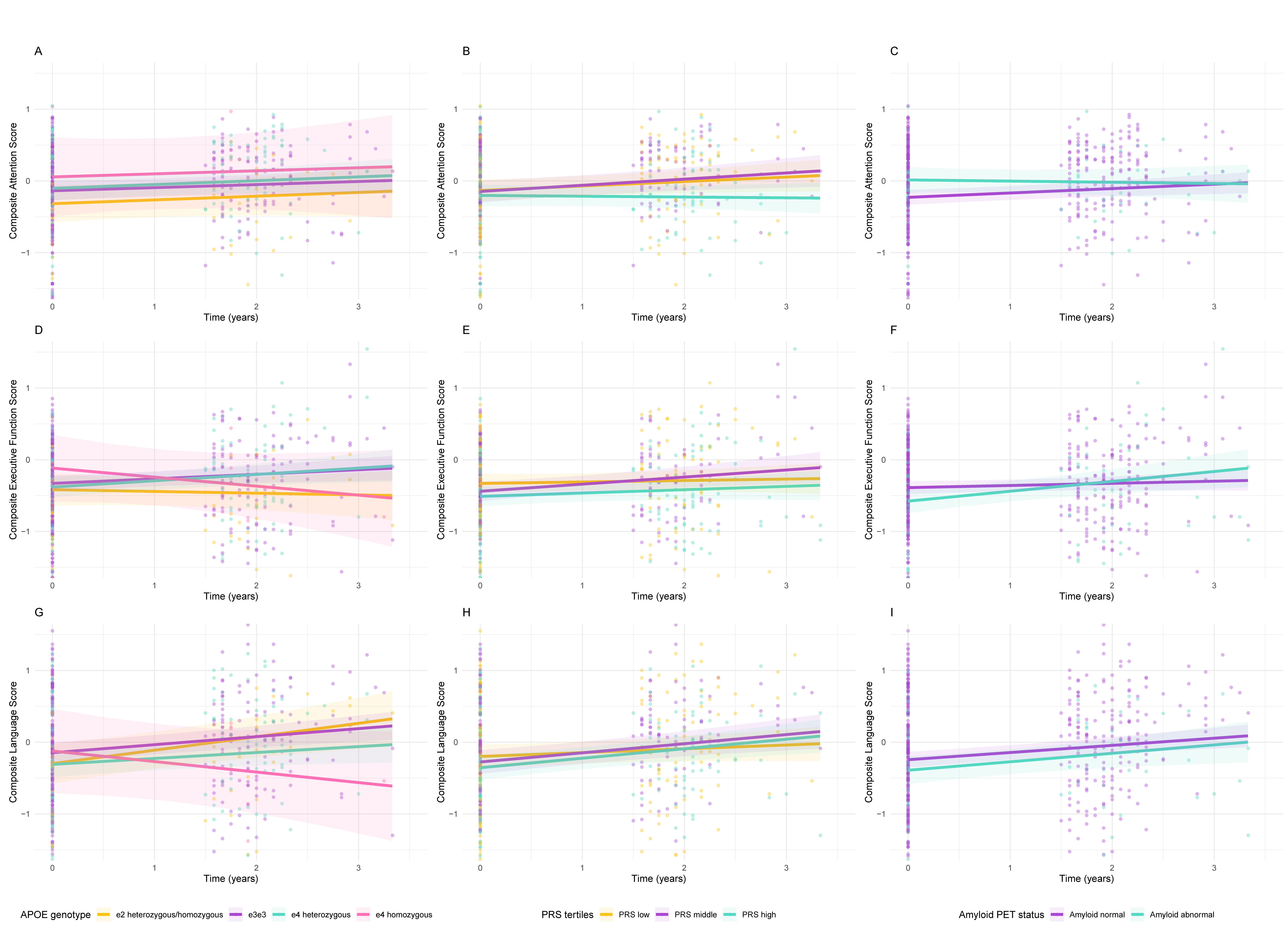

Supplement: Supplementary file 1 — Supplementary Material 1: Figure S1. [file 12883_2022_2925_MOESM1_ESM.png]

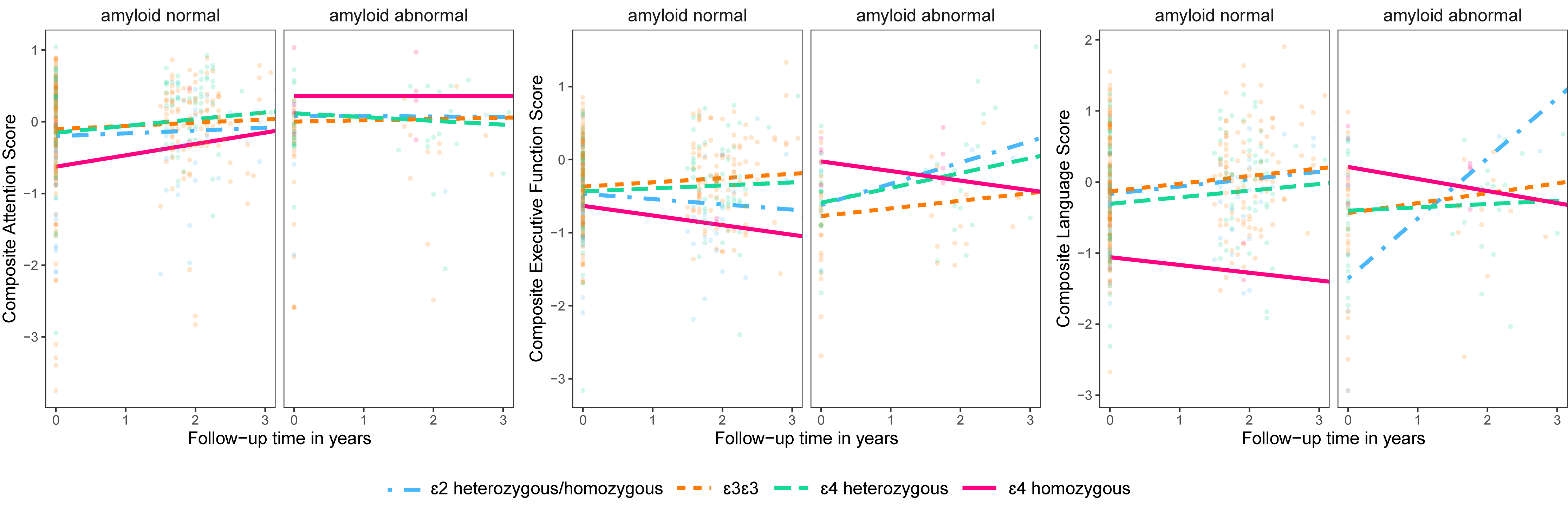

Supplement: Supplementary file 2 — Supplementary Material 2: Figure S2. [file 12883_2022_2925_MOESM2_ESM.png]
