# Supplementary material for: Amyloid-β and APOE genotype predict memory decline in cognitively unimpaired older individuals independently of Alzheimer’s disease polygenic risk score
Source: BMC Neurol. 2022 Dec 15;22:484. doi: 10.1186/s12883-022-02925-6 (PMC9753236; doi:10.1186/s12883-022-02925-6)
Supplement: Supplementary file 3 — Supplementary Material 3: Supplementary Materials. [file 12883_2022_2925_MOESM3_ESM.docx]

**Supplementary Materials**

**Table S1. Genetic variants included in Polygenic Risk Score for AD**

| **SNP** | **Chr** | **Gene** | **A1** | **A2** | **Ref** | **Beta** | **SE** | **Freq A1** |
| --- | --- | --- | --- | --- | --- | --- | --- | --- |
| rs141749679 | 1 | SORT1 | T | C | T | -0.3133 | 0.0702 | 0.9962 |
| rs679515 | 1 | CR1 | T | C | T | 0.1244 | 0.0104 | 0.1877 |
| rs72777026 | 2 | ADAM17 | A | G | A | -0.0563 | 0.0118 | 0.8564 |
| rs17020490 | 2 | PRKD3 | T | C | T | -0.0537 | 0.0115 | 0.8554 |
| rs143080277 | 2 | NCK2 | T | C | T | -0.3915 | 0.0602 | 0.9949 |
| rs6733839 | 2 | BIN1 | T | C | C | 0.1686 | 0.0084 | 0.3891 |
| rs139643391 | 2 | WDR12 | T | TC | TC | -0.063 | 0.0122 | 0.1311 |
| rs10933431 | 2 | INPP5D | C | G | G | 0.0852 | 0.0099 | 0.7657 |
| rs16824536 | 3 | MME | A | G | G | -0.0857 | 0.0185 | 0.0542 |
| rs61762319 | 3 | MME | A | G | A | -0.143 | 0.0255 | 0.9737 |
| rs3822030 | 4 | IDUA | T | G | G | 0.0514 | 0.0083 | 0.5712 |
| rs6846529 | 4 | CLNK | T | C | C | -0.0673 | 0.0091 | 0.7174 |
| rs2245466 | 4 | RHOH | C | G | G | -0.0467 | 0.0091 | 0.6569 |
| rs112403360 | 5 | ANKH | A | T | T | 0.073 | 0.0157 | 0.0727 |
| rs62374257 | 5 | COX7C | T | C | T | -0.0713 | 0.0096 | 0.7705 |
| rs871269 | 5 | TNIP1 | T | C | C | -0.0408 | 0.0088 | 0.3264 |
| rs113706587 | 5 | RASGEF1C | A | G | G | 0.0925 | 0.0133 | 0.1103 |
| rs6605556 | 6 | HLA-DQA1 | A | G | A | 0.0975 | 0.0114 | 0.8387 |
| rs10947943 | 6 | UNC5CL | A | G | G | -0.0529 | 0.0117 | 0.142 |
| rs143332484 | 6 | TREM2 | T | C | C | 0.3346 | 0.0376 | 0.0126 |
| rs75932628 | 6 | TREM2 | T | C | C | 0.8843 | 0.0813 | 0.0031 |
| rs60755019 | 6 | TREML2 | A | G | A | -0.4394 | 0.0842 | 0.9958 |
| rs7767350 | 6 | CD2AP | T | C | C | 0.0629 | 0.0091 | 0.2709 |
| rs785129 | 6 | HS3STS | T | C | T | 0.0443 | 0.0086 | 0.3502 |
| rs6943429 | 7 | UMAD1 | T | C | T | 0.043 | 0.0084 | 0.4205 |
| rs10952097 | 7 | ICA1 | T | C | T | 0.0707 | 0.0138 | 0.1136 |
| rs13237518 | 7 | TMEM106B | A | C | C | -0.0413 | 0.0082 | 0.4115 |
| rs1160871 | 7 | JAZF1 | G | GTCTT | GTCTT | -0.0533 | 0.01 | 0.2223 |
| rs6966331 | 7 | EPDR1 | T | C | T | -0.039 | 0.0085 | 0.3494 |
| rs76928645 | 7 | SEC61G | T | C | C | -0.0762 | 0.0135 | 0.1033 |
| rs7384878 | 7 | SPDYE3 | T | C | C | 0.0775 | 0.0089 | 0.69 |
| rs11771145 | 7 | EPHA1 | A | G | G | -0.0604 | 0.0085 | 0.3476 |
| rs1065712 | 8 | CTSB | C | G | G | 0.1081 | 0.0185 | 0.053 |
| rs73223431 | 8 | PTK2B | T | C | C | 0.0656 | 0.0084 | 0.3694 |
| rs11787077 | 8 | CLU | T | C | T | -0.1002 | 0.0083 | 0.392 |
| rs34173062 | 8 | SHARPIN | A | G | G | 0.1139 | 0.0163 | 0.0814 |
| rs1800978 | 9 | ABCA1 | C | G | C | -0.0682 | 0.0121 | 0.87 |
| rs7912495 | 10 | USP6NL | A | G | A | -0.0572 | 0.0082 | 0.5381 |
| rs7068231 | 10 | ANK3 | T | G | T | -0.0487 | 0.0084 | 0.4026 |
| rs6586028 | 10 | TSPAN14 | T | C | C | 0.0791 | 0.0103 | 0.8036 |
| rs6584063 | 10 | BLNK | A | G | G | 0.1118 | 0.0214 | 0.9565 |
| rs7908662 | 10 | PLEKHA1 | A | G | A | 0.0378 | 0.0081 | 0.5326 |
| rs10437655 | 11 | SPI1 | A | G | G | 0.0566 | 0.0083 | 0.3987 |
| rs1582763 | 11 | MS4A4A | A | G | G | -0.086 | 0.0084 | 0.371 |
| rs3851179 | 11 | EED | T | C | T | -0.1054 | 0.0084 | 0.3584 |
| rs74685827 | 11 | SORL1 | T | G | T | -0.1964 | 0.0303 | 0.9814 |
| rs11218343 | 11 | SORL1 | T | C | T | 0.1654 | 0.0214 | 0.961 |
| rs6489896 | 12 | TPCN1 | T | C | T | -0.073 | 0.0155 | 0.9236 |
| rs17125924 | 14 | FERMT2 | A | G | A | -0.0881 | 0.0142 | 0.9108 |
| rs7401792 | 14 | SLC24A4 | A | G | G | -0.038 | 0.0085 | 0.6291 |
| rs12590654 | 14 | SLC24A4 | A | G | G | -0.0693 | 0.0087 | 0.3279 |
| rs7157106 | 14 | IGH gene cluster | A | G | A | 0.0564 | 0.0107 | 0.3605 |
| rs10131280 | 14 | IGH gene cluster | A | G | G | -0.0653 | 0.0126 | 0.1325 |
| rs8025980 | 15 | SPPL2A | A | G | A | 0.0386 | 0.0085 | 0.6552 |
| rs602602 | 15 | MINDY2 | A | T | T | -0.062 | 0.0091 | 0.2795 |
| rs117618017 | 15 | APH1B | T | C | C | 0.113 | 0.0119 | 0.1439 |
| rs3848143 | 15 | SNX1 | A | G | G | -0.0482 | 0.0099 | 0.7801 |
| rs12592898 | 15 | CTSH | A | G | A | -0.0597 | 0.0123 | 0.1327 |
| rs1140239 | 16 | DOC2A | T | C | C | -0.0592 | 0.0086 | 0.3789 |
| rs889555 | 16 | BCKDK | T | C | C | -0.0555 | 0.0091 | 0.281 |
| rs4985556 | 16 | IL34 | A | C | C | 0.0581 | 0.0128 | 0.1148 |
| rs450674 | 16 | MAF | T | C | T | 0.0451 | 0.0085 | 0.6272 |
| rs12446759 | 16 | PLCG2 | A | G | G | 0.0587 | 0.0084 | 0.597 |
| rs72824905 | 16 | PLCG2 | C | G | C | 0.3065 | 0.0485 | 0.9922 |
| rs16941239 | 16 | FOXF1 | A | T | T | 0.1196 | 0.0256 | 0.0288 |
| rs56407236 | 16 | PRDM7 | A | G | G | 0.1097 | 0.0162 | 0.0693 |
| rs35048651 | 17 | WDR81 | T | TGAG | TGAG | 0.0514 | 0.0102 | 0.2137 |
| rs7225151 | 17 | SCIMP | A | G | G | 0.0856 | 0.0122 | 0.1241 |
| rs2242595 | 17 | MYO15A | A | G | G | -0.0592 | 0.0129 | 0.1117 |
| rs5848 | 17 | GRN | T | C | C | 0.0646 | 0.0092 | 0.2886 |
| rs199515 | 17 | MAPT | C | G | G | 0.0576 | 0.0099 | 0.7806 |
| rs616338 | 17 | ABI3 | T | C | T | 0.2069 | 0.0796 | 0.0122 |
| rs2526377 | 17 | TSPOAP1 | A | G | A | 0.0452 | 0.0082 | 0.5551 |
| rs4277405 | 17 | ACE | T | C | C | 0.0675 | 0.0084 | 0.6163 |
| rs12151021 | 19 | ABCA7 | A | G | A | 0.1055 | 0.0093 | 0.3357 |
| rs149080927 | 19 | KLF16 | G | GC | G | 0.0535 | 0.0092 | 0.4802 |
| rs9304690 | 19 | SIGLEC11 | T | C | C | 0.0458 | 0.0101 | 0.2398 |
| rs587709 | 19 | LILRB2 | T | C | C | -0.0548 | 0.01 | 0.6749 |
| rs1358782 | 20 | RBCK1 | A | G | A | -0.0467 | 0.0098 | 0.246 |
| rs6014724 | 20 | CASS4 | A | G | A | 0.1176 | 0.0145 | 0.9102 |
| rs6742 | 20 | SLC2A4RG | T | C | T | -0.0492 | 0.0104 | 0.221 |
| rs2154481 | 21 | APP | T | C | C | 0.05 | 0.0082 | 0.5236 |
| rs2830489 | 21 | ADAMTS1 | T | C | C | -0.0547 | 0.0091 | 0.2809 |

Genetic variants included in the AD-PRS used. AD-PRSs were calculated based on the 83 genetic variants that showed genome-wide significant (GWS, p<5e-8) evidence of association with AD (Bellenguez C, et al. "New insights into the genetic etiology of Alzheimer’s disease and related dementias." Nature genetics (2022)). The AD-PRS was generated by multiplying the genotype dosage of each risk allele for each variant by its respective weight and then summing across all variants, and were weighted by the effect size from a previous genome-wide association studies on AD. Abbreviations: AD = Alzheimer’s Disease; A1, allele 1; A2, allele 2; Freq A1, frequency of allele 1; Chr = chromosome; Ref = reference allele; SNP, single-nucleotide polymorphisSE = standard error; PRS = polygenic risk score

**Table S2. Baseline and annual change effects of *APOE* genotype with and without ɛ2ɛ4 carriers, PRS and amyloid-β status in memory composite scores**

| Composite memory score | | | | | |
| --- | --- | --- | --- | --- | --- |
|  |  | *APOE genotype without ɛ2ɛ4* | | *APOE genotype with ɛ2ɛ4* | |
| *Model* | *Fixed effects*  *Baseline and interaction effects with time* | *β (SE)* | *p* | *β (SE)* | *p* |
| Model 1 | APOE  APOE × time | 0.0003 (0.07)  -0.064 (0.02) | 0.997  0.002 | -0.02 (0.07)  -0.05 (0.02) | 0.82  0.008 |
| Model 4 | APOE × AD-PRS  APOE × AD-PRS × time | -0.11 (0.72) -0.0008 (0.02) | 0.09  0.97 | -0.13(0.07)  0.002 (0.02) | 0.07  0.94 |
|  | APOE  AD-PRS  APOE × time  AD-PRS × time | -0.01 (0.07)  -0.01 (0.05)  -0.06 (0.02)  -0.04 (0.01) | 0.85  0.83  0.004  0.007 | -0.03 (0.07)  0.002 (0.05)  -0.05 (0.02)  -0.04 (0.01) | 0.66  0.97  0.01  0.008 |
| Model 5 | APOE × Amyloid-β  APOE × Amyloid-β × time | -0.15 (0.15)  -0.11 (0.05) | 0.29  0.02 | -0.11 (0.15)  -0.12 (0.05) | 0.47  0.02 |
|  | Amyloid-β negative group:  APOE  APOE × time  Amyloid-β positive group:  APOE  APOE × time | 0.10 (0.08)  -0.02 (0.02)  -0.07 (0.18)  -0.14 (0.06) | 0.21  0.24  0.68  0.03 | 0.06 (0.08)  -0.02 (0.02)  -0.04 (0.18)  -0.13 (0.06) | 0.44  0.46  0.82  0.03 |

Relationship between *APOE* genotype dosage, AD-PRS, and baseline amyloid-β status and longitudinal memory performance. Linear Mixed Models with subject specific random intercepts and fixed slopes, corrected for age, sex, education and center. *APOE* genotype or PC1-3 were added depending on using *APOE* genotype or AD-PRS as predictor. Amyloid-β status was based on visual read [^18^F]flutemetamol PET standardized uptake value images. Abbreviations: amyloid-β = amyloid-beta; *APOE* = *apolipoprotein* E; p = p value; PRS = polygenic risk score; PC = principal components; SE = standard error.

**Table S3. Baseline and annual change effects of *APOE* genotype, PRS and amyloid-β status in cognitive composite scores**

|  |  | **Attention composite score** | | | **Executive Function composite score** | | | **Language composite score** | | |
| --- | --- | --- | --- | --- | --- | --- | --- | --- | --- | --- |
| *Model* | *Fixed effects*  *Baseline and interaction effects with time* | *β (SE)* | *p* | *pFDR* | *β (SE)* | *p* | *pFDR* | *β (SE)* | *p* | *pFDR* |
| **Model 1** | *APOE*  *APOE* × time | 0.09 (0.07)  0.002 (0.03) | 0.15  0.95 | 0.61  0.95 | 0.03 (0.06)  0.02 (0.03) | 0.66  0.54 | 0.82  0.65 | -0.03 (0.07)  -0.07 (0.03) | 0.73  **0.04** | 0.73  0.12 |
| **Model 2** | AD-PRS  AD-PRS × time | -0.06 (0.05)  0.02 (0.02) | 0.21  0.38 | 0.62  0.81 | 0.03 (0.05)  0.02 (0.02) | 0.46  0.47 | 0.69  0.65 | -0.10 (0.07)  0.02 (0.02) | 0.12  0.38 | 0.28  0.74 |
| **Model 3** | Amyloid-β  Amyloid-β × time | 0.22 (0.10)  -0.08 (0.05) | **0.03**  0.12 | 0.24  0.72 | -0.22 (0.09)  0.10 (0.05) | **0.02**  **0.049** | 0.12  0.36 | -0.16 (0.11)  0.02 (0.05) | 0.13  0.76 | 0.28  0.90 |
| **Model 4** | *APOE* × AD-PRS  *APOE* × AD-PRS × time | -0.05 (0.06)  0.008 (0.03) | 0.42  0.79 | 0.63  0.86 | -0.09 (0.06)  0.02 (0.03) | 0.16  0.42 | 0.32 | -0.17 (0.07)  0.01 (0.03) | **0.02**  0.74 | 0.12  0.90 |
|  | *APOE*  AD-PRS  *APOE* × time  AD-PRS × time | 0.06 (0.06)  -0.04 (0.04)  0.01 (0.03)  -0.02 (0.02) | 0.32  0.36  0.75  0.36 | 0.62  0.62  0.86  0.81 | -0.01 (0.06)  -0.07 (0.04)  0.02 (0.03)  0.01 (0.02) | 0.81  **0.049**  0.48  0.49 | 0.82  0.20  0.65  0.65 | -0.04 (0.07)  -0.06 (0.05)  -0.06 (0.03)  0.02 (0.02) | 0.55  0.21  **0.04**  0.32 | 0.60  0.36  0.12  0.74 |
| **Model 5** | *APOE* × Amyloid-β  *APOE* × Amyloid-β × time | 0.04 (0.14)  -0.04 (0.07) | 0.77  0.56 | 0.77  0.84 | 0.20 (0.13)  -0.10 (0.07) | 0.12  0.18 | 0.29  0.65 | 0.36 (0.15)  -0.21 (0.07) | **0.02**  **0.003** | 0.12  **0.02** |
|  | Amyloid-β negative group:  *APOE*  *APOE* × time  Amyloid-β positive group:  *APOE*  *APOE* × time | 0.04 (0.08)  0.03 (0.03)  0.09 (0.14)  -0.03 (0.07) | 0.61  0.46  0.51  0.72 | 0.73  0.81  0.68  0.86 | 0.02 (0.07)  0.03 (0.04)  0.14 (0.13)  -0.07 (0.07) | 0.82  0.39  0.27  0.34 | 0.82  0.65  0.46  0.65 | -0.07 (0.08)  -0.02 (0.04)  0.27 (0.18)  -0.22 (0.07) | 0.42  0.62  0.14  **0.002** | 0.56  0.90  0.28  **0.02** |
| **Model 6** | AD-PRS × Amyloid-β  AD-PRS × Amyloid-β × time | 0.03 (0.09)  -0.10 (0.05) | 0.72  *0.07* | 0.77  0.72 | 0.04 (0.09)  -0.02 (0.06) | 0.69  0.74 | 0.82  0.74 | 0.06 (0.10)  -0.007 (0.06) | 0.54  0.90 | 0.60  0.90 |
|  | AD-PRS  Amyloid-β  AD-PRS × time  Amyloid-β × time | -0.05 (0.04)  0.20 (0.10)  -0.02 (0.02)  -0.06 (0.05) | 0.27  **0.04**  0.47  0.22 | 0.62  0.24  0.81  0.81 | -0.07 (0.04)  -0.22 (0.09)  0.01 (0.02)  0.10 (0.05) | *0.07*  **0.02**  0.65  *0.06* | 0.21  0.12  0.71  0.36 | -0.05 (0.05)  -0.20 (0.11)  0.02 (0.02)  0.01 (0.06) | 0.25  *0.07*  0.43  0.85 | 0.38  0.28  0.74  0.90 |

Relationship between *APOE* genotype dosage, AD-PRS, and baseline amyloid-β status and longitudinal attention, executive function and language performance. Linear Mixed Models with subject specific random intercepts and fixed slopes, corrected for age, sex, education and center. *APOE* genotype or PC1-3 were added depending on using *APOE* genotype or AD-PRS as predictor. Amyloid-β status was based on visual read [^18^F]flutemetamol PET standardized uptake value images. Abbreviations: amyloid-β = amyloid-beta; *APOE* = *apolipoprotein* E; FDR = False Discovery Rate; p = p value; PRS = polygenic risk score; PC = principal components; SE = standard error.

**Table S4. Baseline characteristics of participants remaining in study and dropouts**

|  | **Overall** | **Participants remaining in study** | **Dropouts** |
| --- | --- | --- | --- |
| *N*  *Manchester cohort, n (%)*  *Amsterdam cohort, n (%)* | 276  77 (27.9)  199 (72.1) | 226  37 (16.4)  189 (83.6) | 50  40 (80)  10 (20) |
| Age, mean (SD) | 74.66 (9.65) | 72.66 (8.95) | 83.70 (7.31) ** |
| Female, n (%) | 174 (63.0) | 134 (59.3) | 40 (80.0) * |
| Years of education, mean (SD) | 11.47 (2.74) | 11.54 (2.72) | 11.19 (2.84) |
| Amyloid-β PET^$^, n (%) | 52 (19.3) | 41 (18.2) | 11 (24.4) |
| *APOE* ɛ4^#^ carrier, n (%) | 84 (30.5) | 77 (34.2) | 7 (14.0) ** |
| *APOE* genotype, n (%) | | | |
| *e2e2* | 2 (0.7) | 2 (0.9) | 0 (0) |
| *e2e3* | 32 (11.6) | 26 (11.5) | 6 (12) |
| *e2e4* | 9 (3.3) | 7 (3.1) | 2 (4) |
| *e3e3* | 157 (56.9) | 120 (53.1) | 37 (74) |
| *e3e4* | 68 (24.6) | 63 (27.9) | 5 (10) |
| *e4e4* | 7 (2.5) | 7 (3.1) | 0 (0) |
| Polygenic Risk Score for AD^^^ (*z* score), mean (SD) | 0.00 (1.00) | 0.03 (1.00) | -0.14 (1.01) |
| MMSE, mean (SD) | *28.87 (1.24)* | *28.94 (1.14)* | 28.52 (1.58) |
| Composite memory (*z* score), mean (SD) | *-0.02 (0.71)* | *0.01 (0.68)* | -0.12 (0.85) |

Differences between participants remaining in study at follow-up and dropouts were assessed using generalized estimating equation models corrected for family relatedness. *p<0.05 **p<0.01 different from participants remaining in study at follow-up. Abbreviations: *APOE* = apolipoprotein E; MMSE = mini mental state examination; Amyloid-β = amyloid beta; t-tau = total-tau; PET = positron emission tomography; p-tau = 181-phosphorylated-tau; SD = standard deviation.

**Figure S1. Effects of *APOE* genotype, AD-PRS and amyloid-β status on changes in cognitive composite scores**

Model-based estimation of longitudinal changes in attention composite score, based on *APOE* genotype (A), polygenic risk score (PRS) for Alzheimer’s Disease (B) and amyloid-β status (C); executive function composite score, based on *APOE* genotype (D), polygenic risk score (PRS) for Alzheimer’s Disease (E) and amyloid-β status (F); language composite score, based on *APOE* genotype (G), polygenic risk score (PRS) for Alzheimer’s Disease (H) and amyloid-β status (I). Neuropsychological tests were *z*-transformed and averaged for the cognitive composite scores. *APOE* genotype dosage variable used as factor for plots: ɛ2 heterozygous/homozygous (*APOE* ɛ2ɛ2:ɛ2ɛ3), ɛ3ɛ3 (*APOE* ɛ3ɛ3), ɛ4 heterozygous (*APOE* ɛ2ɛ4:ɛ3ɛ4) and ɛ4 homozygous (*APOE* ɛ4ɛ4). AD-PRS depicted in tertiles. Amyloid-β status was based on visual read [^18^F]flutemetamol PET standardized uptake value images. Fixed-effect covariates were baseline age, sex, education, center and population substructure. For statistics see Table S2. AD = Alzheimer’s disease; *APOE* = *apolipoprotein* E; PET = positron emission tomography; PRS = polygenic risk score.

**Figure S2. Interaction effects of *APOE* genotype and amyloid-β status with changes in cognitive composite scores**

Interaction effects between *APOE* genotype and amyloid-β status with attention, executive function and language functioning over time. Amyloid-β status was based on visual read [^18^F]flutemetamol PET standardized uptake value images. *APOE* dosage variable used as factor for plots: ɛ2 heterozygous/homozygous (*APOE* ɛ2ɛ2:ɛ2ɛ3, n=34), ɛ3ɛ3 (*APOE* ɛ3ɛ3, n=157), ɛ4 heterozygous (*APOE* ɛ2ɛ4:ɛ3ɛ4, n=77) and ɛ4 homozygous (*APOE* ɛ4ɛ4, n=7). Neuropsychological tests were z-transformed and averaged for the cognitive composite scores. For statistics see Table S2. Abbreviations: *APOE* = *apolipoprotein* E.
